# Clinical and sociodemographic factors associated with late stage cervical cancer diagnosis in Botswana

**DOI:** 10.1186/s12905-021-01402-5

**Published:** 2021-07-06

**Authors:** Tara M. Friebel-Klingner, Rebecca Luckett, Lisa Bazzett-Matabele, Tlotlo B. Ralefala, Barati Monare, Mercy Nkuba Nassali, Doreen Ramogola-Masire, Memory Bvochora, Nandita Mitra, Douglas Wiebe, Timothy R. Rebbeck, Anne Marie McCarthy, Surbhi Grover

**Affiliations:** 1 Department of Biostatistics, Epidemiology and Informatics, Perelman School of Medicine, University of Pennsylvania, Philadelphia, PA USA; 2 Botswana-University of Pennsylvania Partnership, Gaborone, Botswana; 3 Department of Obstetrics and Gynecology, Faculty of Medicine, University of Botswana, Gaborone, Botswana; 4 Department of Obstetrics and Gynecology, Beth Israel Deaconess Medical Center, Boston, MA USA; 5 Department of Obstetrics and Gynecology, Princess Marina Hospital, Gaborone, Botswana; 6 Department of Oncology, Princess Marina Hospital, Gaborone, Botswana; 7 Department of Obstetrics and Gynecology, Yale University, New Haven, CT USA; 8 Dana-Farber Cancer Institute and Harvard TH Chan School of Public Health, Boston, MA USA; 9 Department of Radiation Oncology, University of Pennsylvania, Philadelphia, PA USA

**Keywords:** Cervical cancer, Botswana, Late-stage, HIV

## Abstract

**Background:**

Cervical cancer is the leading cause of female cancer mortality in Botswana with the majority of cervical cancer patients presenting with late-stage disease. The identification of factors associated with late-stage disease could reduce the cervical cancer burden. This study aims to identify potential patient level clinical and sociodemographic factors associated with a late-stage diagnosis of cervical cancer in Botswana in order to help inform future interventions at the community and individual levels to decrease cervical cancer morbidity and mortality.

**Results:**

There were 984 women diagnosed with cervical cancer from January 2015 to March 2020 at two tertiary hospitals in Gaborone, Botswana. Four hundred forty women (44.7%) presented with late-stage cervical cancer, and 674 women (69.7%) were living with HIV. The mean age at diagnosis was 50.5 years. The association between late-stage (III/IV) cervical cancer at diagnosis and patient clinical and sociodemographic factors was evaluated using multivariable logistic regression with multiple imputation. Women who reported undergoing cervical cancer screening had lower odds of late-stage disease at diagnosis (OR: 0.63, 95% CI 0.47–0.84) compared to those who did not report screening. Women who had never been married had increased odds of late-stage disease at diagnosis (OR: 1.35, 95% CI 1.02–1.86) compared to women who had been married. Women with abnormal vaginal bleeding had higher odds of late-stage disease at diagnosis (OR: 2.32, 95% CI 1.70–3.16) compared to those without abnormal vaginal bleeding. HIV was not associated with a diagnosis of late-stage cervical cancer. Rural women who consulted a traditional healer had increased odds of late-stage disease at diagnosis compared to rural women who had never consulted a traditional healer (OR: 1.61, 95% CI 1.02–2.55).

**Conclusion:**

Increasing education and awareness among women, regardless of their HIV status, and among providers, including traditional healers, about the benefits of cervical cancer screening and about the importance of seeking prompt medical care for abnormal vaginal bleeding, while also developing support systems for unmarried women, may help reduce cervical cancer morbidity and mortality in Botswana.

## Introduction

Cervical cancer affects women across the globe, with a disproportionately higher burden of morbidity and mortality in low- and middle-income countries (LMICs) [1]. In 2018, more than 80% of the 311,000 cervical cancer deaths worldwide occurred in LMICs, predominantly in sub-Saharan Africa (SSA) [1]. Cervical cancer is classified as a human immunodeficiency virus (HIV)-related malignancy, further contributing to the increasing cancer burden in SSA countries with a high prevalence of HIV [2]. Additionally, late-stage cervical cancer at diagnosis is significantly associated with increased cervical cancer mortality, and approximately 68% of cervical cancers are diagnosed at a late stage in SSA [3]. Thus, it is paramount to detect cervical cancer at an earlier, more treatable stage in order to significantly reduce cervical cancer deaths [4, 5].

Cervical cancer screening aims to prevent invasive cancer [6–10]. Other clinical and sociodemographic factors have been associated with late-stage cervical cancer at diagnosis, particularly in LMICS, including abnormal vaginal bleeding [6, 7, 11, 12], age at diagnosis [9, 13–15], marital status [6, 15–18], and living in a rural area [7, 15, 16, 18, 19]. In addition, the practice of traditional healers has been shown to be associated with an increase in late-stage cervical cancer at diagnosis [20] and as a barrier to cervical cancer care in low-resource settings [21].

In Botswana, an upper-middle-income country in SSA, cervical cancer is the leading cause of female cancer deaths [22, 23]. However, there remains a dearth of information regarding the demographics and clinical factors that contribute to late-stage cervical cancer at diagnosis among women from SSA. Botswana also has a high prevalence of HIV, with 25.1% of females between 15–49 years of age living with HIV in 2019 [24]. In recent decades, with the growing burden of HIV-related cancers [25], the Botswana Ministry of Health and Wellness (MOHW) has prioritized reducing the cervical cancer burden by adapting American Society of Clinical Oncology (ASCO) resource stratified screening strategies for its citizens, with the majority of cervical cancers being detected through loop electrosurgical excision procedure or visual inspection with acetic acid [26–29]. Despite these efforts by the Botswana MOHW, approximately 50% of cervical cancers are diagnosed at a late stage [6, 30].

This study aims to identify potential clinical and sociodemographic factors associated with a late-stage diagnosis of cervical cancer in Botswana in order to help inform future interventions at the community and individual levels aimed at decreasing cervical cancer morbidity and mortality.

## Methods

### Study participants

We abstracted data from questionnaires administered during the initial consult visit and medical records for women with invasive cervical cancer who had consented to participate in research studies [30, 31] at Princess Marina Hospital (PMH) and Gaborone Private Hospital (GPH), two tertiary referral hospitals in the capital city of Gaborone, between January 2015 and March 2020 [30, 31]. GPH houses the sole chemo-radiation facility in the country. Women diagnosed at either the public hospital PMH or GPH, are treated at GPH, and their treatment is covered under the government health care system. Women were eligible for this analysis if they were over the age of 18 years, not pregnant, and diagnosed with cervical cancer. Women were excluded if they were diagnosed with cervical carcinoma in situ or if they had recurrent disease.

### Covariates

Data collected at the time of cancer diagnosis included patient/sociodemographic and clinical factors (i.e., age, marital status, place of residence, history of cervical cancer screening, ever/never visit with a traditional doctor and/or natural healer, presence of abnormal vaginal bleeding (including post-coital bleeding/bleeding after vaginal intercourse), HIV status, and utilization of antiretroviral therapy (ART)). Additional clinical data was abstracted from medical records regarding clinical factors, such as stage, pathology, and CD4 count. Place of residence was characterized as urban or rural based on the sub-district of the participant’s reported residence [32].

Cervical cancer stage at diagnosis was based on the International Federation of Gynecology and Obstetrics (FIGO) staging system [33, 34]. FIGO cervical cancer stages were dichotomized as early-stage (I–II) and late-stage (III–IV).

### Statistical analyses

Descriptive statistics for each variable of interest were examined for the entire study sample, and by cervical cancer stage at diagnosis (early vs. late). Differences between early- and late-stage disease were examined using Pearson’s chi-squares test for categorical variables, Fisher’s exact test for small sample sizes, and Student t-tests for continuous variables. Multivariable logistic regression was used to examine potential risk factors associated with late- versus early-stage disease. Variables included in the multivariable model were determined based on purposeful selection, review of the literature, and clinical relevance. We assessed missing data, patterns, and reasons for missing data. To account for missing data, we performed multiple imputation with chained equations (MICE) for the multivariable logistic regression model, assuming data were missing at random [35–37]. We also conducted complete case analyses (results not shown). We further investigated interactions between HIV status and age and HIV and screening history. We also investigated the use of traditional healers in rural and urban areas using univariate logistic regression. Additionally, because cervical cancer is an AIDS-defining malignancy, we examined clinical and sociodemographic differences between women living with HIV (WLWH) and women without HIV, and performed multivariable analyses stratified by HIV status. For the analysis of WLWH, CD4 count and ART use were included as potential risk factors. All statistical analyses were conducted using STATA 16, and p-values < 0.05 were considered statistically significant.

### Ethics approval

This study, “Treatment and Outcomes of Patients Presenting with Cancer in Botswana,” was approved by the University of Pennsylvania as part of the Botswana-University of Pennsylvania Partnership (IRB: 820159 IRB#7 Penn) and by the Ministry of Health and Wellness of the Republic of Botswana (HPDME 13/18/1).

## Results

### Patient characteristics

Between January 2015 and March 2020, 1,007 women were diagnosed with cervical cancer. We excluded 16 women with prior cervical carcinoma in situ and seven women with recurrent disease, resulting in the inclusion of 984 women in this study. Sociodemographic and clinical characteristics are shown in Table 1. Four hundred forty (44.7%) of the women included in the study were diagnosed with late-stage cervical tumors. The mean age at diagnosis was 50.5 years (range 22.4–95.2), 21.0% (n = 206) lived in urban areas, 65.7% (n = 646) had never been married, 57.4% (n = 539) reported previous cervical cancer screening, 69.7% (n = 674) were WLWH, and 10.1% (n = 95) reported ever having a visited with a traditional healer. Abnormal vaginal bleeding was reported in 73.2% (n = 720) of women, and 87.3% (n = 835) of the cervical cancers were squamous cell carcinoma (SCC) pathology. Women diagnosed at a late stage were less likely to report prior screening (50.8% vs. 63.2%, p < 0.001) and were also more likely to report abnormal vaginal bleeding (82.3% vs. 66.3%, p < 0.001).

**Table 1:** Clinical and demographic characteristics of the study population by early- versus late-stage cervical cancer diagnosis

Variable	Study population		Early stage		Late stage		P-value**
	N	%	N	%	N	%	
	984	100	501	50.9	440	44.7	
Age category							
< 30	13	1.3	7	1.4	6	1.4	0.88
≥ 30–40	188	19.1	98	19.6	83	18.9	
≥ 40–50	369	37.5	191	38.2	159	36.1	
≥ 50–60	183	18.6	98	19.6	83	18.9	
≥ 60–70	148	15.1	66	13.2	70	15.9	
≥ 70	82	8.3	40	8.0	39	8.9	
Residence							
Rural	775	79.0	390	78.0	359	81.8	0.15
Urban	206	21.0	110	22.0	80	18.2	
Marital status							
Never married/single	646	65.7	315	63.0	307	69.8	0.08
Married	226	23.0	124	24.8	89	20.2	
Divorced	13	1.3	10	2.0	3	0.7	
Widowed	98	10.0	51	10.2	41	9.3	
Previous cervical cancer screening							
Never screened	400	42.6	177	36.8	205	49.2	< 0.001*
Screened	539	57.4	304	63.2	212	50.8	
Visit with a natural/traditional healer							
No	845	89.9	443	90.8	378	88.3	0.22
Yes	95	10.1	45	9.2	50	11.7	
HIV							
Negative	293	30.3	154	31.2	128	29.6	0.59
Positive	674	69.7	340	68.8	305	70.4	
FIGO stage							
I	166	17.6	166	33.1	–	–	–
II	335	35.6	335	66.9	–	–	
III	350	37.2	–	–	350	79.5	
IV	90	9.6	–	–	90	20.5	
Pathology							
SCC	835	87.3	422	86.1	384	89.7	0.29
Adenocarcinoma	66	6.9	41	8.4	22	5.1	
Invasive ductal	4	0.4	1	0.2	3	0.7	
Other	40	4.2	20	4.1	15	3.5	
Unknown/other	12	1.2	6	1.2	4	0.9	
Abnormal vaginal bleeding							
Not reported	264	26.8	169	33.7	78	17.7	< 0.001*
Reported	720	73.2	332	66.3	362	82.3	

*p < 0.05

**p-value for differences between early stage and late stage

### Factors associated with late-stage cervical cancer at diagnosis

Table 2 displays factors significantly associated with late-stage cervical cancer at diagnosis in the imputed multivariable model. MICE was performed to account for missing data for the variables: stage at diagnosis (n = 43), age (n = 1), place of residence (n = 3), marital status (n = 1), screening history (n = 45), HIV status (n = 17), and visit with a traditional healer (n = 27). Never being married (OR: 1.35, 95% CI 1.02–1.86) and experiencing abnormal vaginal bleeding (OR: 2.32, 95% CI 1.70–3.16) had an increased odds of late-stage cervical cancer at diagnosis, while previous cervical cancer screening was associated with decreased odds of late-stage cervical cancer at diagnosis (OR: 0.65, 95% CI 0.49–0.85). Results from the complete case analysis were analogous to the MICE results. No significant interactions were observed between HIV status and age or between HIV status and screening history.

**Table 2:** Imputed multivariable analysis of factors associated with late-stage cervical cancer

Late stage at diagnosis	Adjusted odds ratio	95% CI	P-value
Age	1.01	1.00–1.03	0.067
Urban versus rural residence	0.78	0.55–1.10	0.153
Never married/single versus married/widowed/divorced	1.35	1.02–1.86	0.044*
Cervical cancer screening versus never screened	0.65	0.49–0.85	0.002*
Visit with a natural healer (Yes/No)	1.19	0.77–1.84	0.434
HIV seropositive versus seronegative	1.37	0.97–1.93	0.077
Abnormal vaginal bleeding (Yes/No)	2.32	1.70–3.16	< 0.001*

*95% CI* 95% confidence interval

*p < 0.05

There were no significant associations between living in an urban residence versus living in a rural residence and having ever visited a traditional healer and having late-stage cervical cancer at diagnosis in the multivariable model. However, in our cohort, more rural women than urban women (11% vs. 5%) had consulted with a traditional healer. Table 3 shows the association of presenting with late- versus early-stage cervical cancer at diagnosis when visiting a traditional healer among women living in a rural residence (OR: 1.61, 95% CI 1.02–2.55). This increased probability was not observed among women living in an urban area when visiting a traditional healer.

**Table 3:** Visit with a traditional healer in a rural setting in women with late- versus early-stage cervical cancer

	Early Stage	Late Stage	OR	95% CI	P-value
Visit with a traditional healer	35 (9%)	49 (14%)	1.61	1.02–2.55	0.043*
No visit with a traditional healer	346 (91%)	301 (86%)			

*OR* odds ratio, *95% CI* 95% confidence interval

*p < 0.05

## Characteristics by HIV status

Table 4 shows patient characteristics by HIV status. Of the 984 cervical cancer cases, the HIV status of 967 (98.3%) women was known. Women whose HIV status was unknown (n = 17) were excluded from the HIV stratified analyses. WLWH comprised 69.7% (n = 674) of the study population and were significantly younger than women without HIV (45.8 years vs. 60.5 years, p < 0.001). WLWH were also more likely to live in urban areas (24.0% vs. 14.7%, p = 0.001), were more likely to have never been married (74.0% vs. 47.8%, p < 0.001), and were more likely to have been screened for cervical cancer (61.8% vs. 48.4%, p < 0.001) than women without HIV. In addition, WLWH were more likely to have SCC pathology than women without HIV (88.9% vs. 83.7%, p = 0.020). Among the WLWH, 78.6% (n = 429) had a CD4 cell count > 250 cells/mm^3^ at diagnosis, and 96.2% (n = 640) reported being on ART.

**Table 4:** Clinical and demographic characteristics of the study population by HIV status

Variable	HIV negative		HIV positive		p-value
	N	%	N	%	
	293	30.3	674	69.7	
Age categories					
< 30	2	0.7	11	1.6	< 0.001*
≥ 30–40	23	7.9	165	24.5	
≥ 40–50	44	15.1	324	48.1	
≥ 50–60	60	20.5	119	17.7	
≥ 60–70	98	33.6	47	7.0	
≥ 70	65	22.3	8	1.2	
Residence					
Rural	250	85.3	511	76.0	< 0.001*
Urban	43	14.7	161	24.0	
Marital status					
Never married/single	140	47.8	498	74.0	< 0.001*
Married	91	31.1	132	19.6	
Divorced	3	1.0	9	1.3	
Widowed	59	20.1	34	5.1	
Previous cervical cancer screening					
Never screened	143	51.6	248	38.2	< 0.001*
Screened	134	48.4	401	61.8	
Visit with a traditional healer					
No	250	87.7	599	90.9	0.140
Yes	35	12.3	60	9.1	
FIGO Stage					
I	48	17.0	115	17.8	0.83
II	106	37.6	225	34.9	
III	100	35.5	245	38.0	
IV	28	9.9	60	9.3	
Pathology					
SCC	239	83.9	583	88.9	0.022*
Adenocarcinoma	31	10.9	33	5.0	
Invasive ductal	1	0.4	3	0.5	
Other	9	3.2	30	4.6	
Vascular invasion	0	0	1	0.2	
Unknown	5	1.8	6	0.9	
Abnormal vaginal bleeding					
Not reported	68	23.2	192	28.5	0.089
Reported	225	76.8	482	71.5	
CD4					
< 250 cells/mm^3^	–	–	117	21.4	–
≥ 250 cells/mm^3^	–	–	429	78.6	
ART					
No	–	–	25	3.8	–
Yes	–	–	640	96.2	

*p < 0.05

Table 5 shows the results of the multivariable logistic regression models by HIV status. Among the WLWH, prior cervical cancer screening showed decreased odds with late-stage disease at diagnosis (OR: 0.61, 95% CI 0.44–0.86), and increased odds with previous abnormal bleeding symptoms (OR: 2.10, 95% CI 1.46–3.01). Among women without HIV, factors associated with higher odds of late-stage disease at diagnosis included increasing age (OR: 1.02, 95% CI 1.00–1.14; p = 0.041) and abnormal vaginal bleeding (OR: 3.06, 95% CI 1.52–5.71). Results of complete case analyses by HIV status were similar to the imputed results.

**Table 5:** Imputed multivariable subgroup analyses of women living with HIV and women without HIV

Late stage at diagnosis	Adjusted odds ratio	95% CI	P-value	Adjusted odds ratio	95% CI	P-value
	WLWH n = 674			Women without HIV n = 293		
Age	1.01	0.99–1.02	0.480	1.02	1.00–1.04	0.041*
Urban vs. rural residence	0.79	0.54–1.16	0.234	0.88	0.43–1.81	0.730
Never married/single vs. married/widowed/divorced	1.23	0.85–1.79	0.273	1.59	0.94–2.70	0.087
Cervical cancer screening vs. never screened	0.61	0.44–0.86	0.004*	0.80	0.48–1.35	0.413
Visit with a traditional healer (Ye/No)	0.95	0.55–1.65	0.864	1.52	0.71–3.26	0.276
Abnormal vaginal bleeding (Yes/No)	2.10	1.46–3.01	< 0.001*	3.06	1.52–5.71	0.001*
CD4 count < 250 vs. ≥ 250	1.15	0.73–1.79	0.546			
Anti-retroviral treatment (Yes/No)	1.11	0.46–2.65	0.821			

*95% CI* 95% confidence interval

*p < 0.05

## Discussion

This large study of women with cervical cancer in Botswana aimed to identify potential clinical and sociodemographic factors associated with a late-stage diagnosis of cervical cancer in Botswana. Our study showed that prior cervical cancer screening was associated with decreased odds of having late-stage cervical cancer at diagnosis, whereas experiencing abnormal vaginal bleeding and having never been married were associated with an increased odds of having late-stage cervical cancer at diagnosis. Having HIV was not associated with having late-stage cervical cancer at diagnosis. Furthermore, results suggested that women living in rural areas who visited a traditional healer were more likely to be diagnosed with late-stage cervical cancer.

Screening has been shown to lead to an earlier diagnosis of cervical cancer in high-income countries with established screening programs, and screening has also been shown to be effective in low resource settings [7, 9, 38]. With the growing burden of HIV-related cancers in recent decades [25], the Botswana MOHW has prioritized reducing the cervical cancer burden through implementing and supporting a national cervical cancer screening program as part of the HIV care continuum [23, 27, 28, 39, 40]. A prior retrospective study by Nassali et al. (2018) reviewed 149 cervical cancer patients admitted to PMH from August 2016 to February 2017, which may include some overlap with our study sample. In that study, Nassali et al. defined late-stage cervical cancer as FIGO stage IB2-IVB, and found an increased odds of presenting with late-stage tumors in patients not previously screened for cervical cancer. Additionally, two qualitative studies in Botswana [41, 42] have shown that lack of knowledge regarding the benefits of screening for cervical cancer can delay diagnosis. Our study provides further evidence supporting the finding that screening decreases the odds of presenting with late-stage cervical cancer at diagnosis when implemented in a low resource setting.

Early-stage asymptomatic cervical cancer can be detected through screening, but in the absence of screening, patients with cervical cancer can present with clinical symptoms including abnormal vaginal bleeding and post-coital bleeding [34]. Reports of symptomatic bleeding and its association with late-stage disease at diagnosis in low resource settings have been inconsistent in the literature [6, 7, 11, 12]. Studies have shown an increased risk [6], no association [7], and a decreased risk [11, 12]. In Nepal, a decreased risk for having late-stage cervical cancer at diagnosis was noted if the symptoms of bleeding were reported first to the woman’s husband, who might encourage his wife to seek medical care. In Morocco, the decreased association between having late-stage cervical cancer at diagnosis and having symptomatic bleeding was hypothesized to be due to understanding the severity of bleeding as a cervical cancer symptom and seeking medical care without delay. Two studies in Botswana [20, 43] showed that the perception of symptom severity was related to having advanced stage cervical cancer at diagnosis and to having a delay in health seeking behavior. In our study, reporting gynecological bleeding symptoms, including previous abnormal vaginal bleeding and/or post-coital bleeding, was associated with a two-fold increase in the odds of presenting with late-stage cervical cancer. These results support increasing awareness regarding abnormal vaginal bleeding and post-coital bleeding as indications of cervical cancer and emphasizing the need to seek medical care as soon as possible. In addition, these findings support screening asymptomatic women to be able to diagnose cervical cancer at an early stage.

In our study, there was no significant difference between WLWH and women without HIV with regard to their likelihood of presenting with advanced stage cervical cancer. Some studies have recognized HIV as a risk factor for late-stage cervical cancer at diagnosis [16, 19], yet other studies [6, 44] have reported no association with HIV and late-stage cervical cancer at diagnosis. The role of HIV in the diagnosis of late-stage cervical cancer remains unclear and should be investigated further. When comparing WLWH and women without HIV in our cohort, the WLWH were younger, were more likely to have undergone cervical cancer screening, had more often lived in urban areas, and were more likely to be married or to have been married than women without HIV. Also, in the subgroup analyses, associations with late-stage cervical cancer at diagnosis differed between the two subgroups of WLWH versus women without HIV. Increasing age was significantly associated with late-stage cervical cancer at diagnosis in women without HIV, but not in WLWH. WLWH with a history of cervical cancer screening had lower odds of presenting with late-stage cervical cancer at diagnosis; however cervical cancer screening was not significantly associated with late-stage cervical cancer at diagnosis in women without HIV. In Botswana, cervical cancer screening programs have been implemented as part of the HIV care continuum for women, making it is plausible that women without HIV do not access screening services to the same extent as WLWH. Therefore, increasing screening services among women without HIV could reduce the prevalence of late-stage cervical cancer at diagnosis for these women.

Our study saw a decrease in late-stage cervical cancer cases among women who had been married. Similarly, a study from Nepal [11] noted that married women with symptomatic bleeding were less likely to present with a late-stage cervical cancer because husbands may encourage their wives to seek medical care. A study by Ibrahimi and Pinheiro (2017) in the United States reported that being married was an independent predictor of a more favorable prognosis of cervical cancer [45]. While some studies have identified being unmarried as a risk factor for having late-stage cervical cancer at diagnosis [6, 17, 18], other studies have found no such association [15, 16]. Reasons for this association are unclear. Future studies investigating differences in financial, emotional, and sociocultural marital structures and the impact on prompt cancer diagnosis are warranted. These inconsistencies could be attributed to differences among the sociocultural marital structures and support systems across countries, which need to be explored further.

Areas of residence may also impact health care. For example, living in a rural area versus living in an urban area has been investigated as a potential contributing factor for women with late-stage cervical cancer at diagnosis [7, 15, 16, 18, 19]. A study in Sudan reported that an increased risk for having late-stage cervical cancer at diagnosis was associated with living in a rural setting versus living in an urban setting [15], but this result was not seen in our cohort, nor was any such association reported in other studies in Ghana [7], Florida [18], Ethiopia [19], or SSA [15]. The use of traditional healers has been shown to be associated with late-stage cervical cancer at diagnosis in Botswana [20] and as a barrier to cervical cancer care in Uganda [21]. In Botswana, over 95% of traditional healers live in rural areas [46], and thus women living in a rural area may be more likely to consult with traditional healers as their first choice for healthcare. In our study, when examining only women living in a rural area, those who visited a traditional healer had a higher odds of presenting with late-stage cervical cancer. Points of intervention in rural areas could include educating traditional healers to recognize symptoms of cervical cancer in order to facilitate a referral for diagnosis and treatment.

This large study investigating late-stage cervical cancer at diagnosis in Botswana includes detailed demographic and clinical information on patients in Botswana collected over a five-year period, but it does have several potential limitations and challenges. It is important to note that, due to the cross-sectional study design, no decisive conclusions can be made about the temporality or causality among the study variables and late stage diagnosis. Patients were enrolled at PMH, a public tertiary hospital with oncology services, and at GPH, a private tertiary hospital with the only chemo-radiation oncology center in Botswana; thus, all patients who need radiotherapy should be sent to GPH. We were unable to account for patients who were not diagnosed with or treated for cervical cancer outside of these two facilities. In addition, the study collected data at the time of diagnosis and is therefore subject to recall bias, social desirability bias, and potential unmeasured confounding and missing data. Unfortunately, due to the retrospective nature of the study, we lacked important information and were unable to account for confounders including education level, knowledge and awareness of cervical cancer, and cervical cancer screening. To account for any bias due to missing data, we conducted MICE for the primary analysis [35–37]. Results using MICE were similar to the complete case analysis. Although our findings represent a large proportion of cervical cancer cases in Botswana, they do not represent all cervical cancer patients in Botswana and are not generalizable to the entire country.

## Conclusion

Our results highlight patient level factors associated with late-stage cervical at diagnosis and indicate potential areas for intervention to mitigate the cervical cancer burden in Botswana. Our findings show that cervical cancer screening for women in Botswana is associated with the early detection of cervical cancer, particularly in women with HIV. Future efforts to include women without HIV and women who have not been married in cervical cancer screening efforts could result in the earlier detection of cervical cancer in these groups. Future early cervical cancer detection efforts should emphasize cancer symptom awareness and early detection through cervical cancer screening, and should also include traditional healers in the cancer care continuum.

## Data Availability

The datasets generated and/or analyzed during the current study are not publicly available due identifying information, but are available from the corresponding author on reasonable request.
